# Circulating mir-200c and mir-33a may be used as biomarkers for predicting high fructose corn syrup-induced fatty liver and vitamin D supplementation-related liver changes

**DOI:** 10.55730/1300-0144.5483

**Published:** 2022-09-09

**Authors:** Alpaslan TANOĞLU, Eylem ÇAĞILTAY, Esra Güzel TANOĞLU, Aylin GÖKHAN, Cansın ŞİRİN, Türker ÇAVUŞOĞLU, Soner YEŞİLBAŞ

**Affiliations:** 1 Department of Gastroenterology, Sancaktepe Şehit Prof. Dr. İlhan Varank Training and Research Hospital,University of Health Sciences, İstanbul, Turkey; 2 Department of Endocrinology and Metabolism, Sultan Abdulhamid Han Training and Research Hospital,University of Health Sciences, İstanbul, Turkey; 3 Department of Molecular Biology and Genetics, Institution of Hamidiye Medical Sciences,University of Health Sciences, İstanbul, Turkey; 4 Department of Histology and Embryology, Faculty of Medicine, Ege University, İzmir, Turkey; 5 Department of Histology and Embryology, Faculty of Medicine, İzmir Bakırçay University, İzmir, Turkey; 6 Department of Biochemistry, Zeynep Kamil Training and Research Hospital, University of Health Sciences, İstanbul, Turkey

**Keywords:** Fatty liver, vitamin D_3_, microRNA, biomarker, rat

## Abstract

**Background/aim:**

Nonalcoholic fatty liver is one of the most common forms of liver disease and role of microRNAs (miRNAs) on this illness is currently unclear. It was aimed to evaluate the predictive role of circulating miR-33a and mir-200c on high fructose corn syrup (HFCS)-induced fatty liver and vitamin D_3_ supplementation-related hepatic changes.

**Materials and methods:**

Twenty-four rats were randomized into three groups: sham (n = 8), experimental fatty liver group (n = 8), and fatty liver + vitamin D_3_ supplementation group (n = 8). In the treatment group, 10 μg/kg/week of vitamin D_3_ was given by orogastric tube weekly for 4 weeks in addition to a high fructose diet. Serum AST, ALT, TNF-α, and MDA levels were tested. Liver tissue samples were examined using hematoxylin/eosin, periodic acid-Schif (PAS) and Masson’s Trichrome staining. Circulating miR-33a and mir-200c were quantified by qRT-PCR method. Moreover, in silico analyses were accomplished.

**Results:**

In the vitamin D_3_ group, results of biochemical parameters were significantly different than those of the fatty liver group (p < 0.001). Moreover, significant differences in serum levels of circulating miR-33a and mir-200c were identified among all groups (p < 0.05). Finally, more favorable histopathological changes were noticed in the vitamin D_3_ supplementation group. The expressions of Ki-67 were also considerably reduced in the vitamin D_3_ group. According to KEGG pathway analysis, mir-33a and mir-200c were found to play a common role in the Hippo signaling pathway, lysine degradation, and protein processing.

**Conclusion:**

Our current experimental fatty liver study showed that, in a specified dose, vitamin D_3_ supplementation could alleviate adverse undesirable hepatic effects of HFCS via miR-33a and mir-200c.

## 1. Introduction

Metabolic syndrome, obesity, and fatty liver prevalence have increased in the last few decades all over the world. The lack of physical activity and modern western-style diet are the two major factors for this epidemic [1, 2]. A major component of this epidemic, nonalcoholic fatty liver disease (NAFLD) is defined as more than 5% triglyceride accumulation in the liver that can lead to steatosis and increased inflammation in some cases, resulting in nonalcoholic steatohepatitis in (NASH) and eventually in cirrhosis and hepatocellular carcinoma [1–3]. Dietary factors have been postulated as having roles in development of hepatic steatosis; however, these factors should be investigated in a detailed manner in order to develop curative therapeutic options [2,3]. In addition to the individual differences, the variability in the eating habits and weighted food products resulting from ethnic and cultural variation may determine these consequences [4]. As a feature of modern western-style diet, the increase in the consumption of high-fructose corn syrup (HFCS) and other corn products is currently under debate in terms of its association with epidemic of NAFLD [5].

Currently, vitamin D deficiency is common in Western countries, particularly in the northern residential areas. The prevalence of vitamin D deficiency is about 40%, ranging from 15% and 80% in previous studies [6, 7]. This variation is mainly determined by the racial differences. Interestingly, even in people living in sunny countries in the Mediterranean region, vitamin D deficiency is sometimes more common than that seen in northern European countries [6, 7] . In addition, in recent studies, vitamin D deficiency is not only associated with calcium-phosphorus metabolism but also is associated with the metabolic syndrome, insulin resistance, and NAFLD. Moreover, determining the efficacy of vitamin D supplementation on fatty liver tissue is still an important research topic [8, 9].

MicroRNAs are short noncoding RNA sequences of approximately 18–22 nucleotides in length that regulate gene expression [10]. The presence of some deregulated miRNAs in NAFLD has been reported and most of them are known to be effective in lipid and cholesterol metabolism. In addition, many miRNAs have been shown to regulate insulin resistance, oxidative stress, and inflammation [11], which are considered critical components of NAFLD. miRNAs act as posttranscriptional regulators and again miRNAs can target hundreds of different genes. In addition, genes can be targeted by many different miRNAs. In recent years, it has been suggested that various miRNAs may have oxidative stress, apoptosis, and endoplasmic reticulum regulatory effects in liver tissue. It has been scientifically proven that miRNAs perform many important functions in various physiological and pathological processes occurring in the liver [10–11]. There is insufficient information about effects of vitamin D_3_ supplementation on fibrogenic miRNAs that play a role in fatty liver. On the other hand, modulatory role of vitamin D_3_ on miRNAs are another important topic of investigation [12].

It has been reported that mir-200c may contribute to hepatic lipid metabolism. In addition, its expression levels are associated with hepatic steatosis and hepatic inflammation [13] . On the other hand, it has been postulated that mir-33a is dysregulated in high-fat-diet-induced fatty liver disease and affect disease course [14]. However, there is no study examining the relationship between vitamin D_3_ supplementation and these two circulating miRNAs in HFCS induced fatty liver model. Moreover, investigating these two circulating miRNAs as candidate biomarker for predicting NAFLD and also fatty liver treatment success is an inevitable concern.In this study, it was aimed to evaluate the predictive role of circulating miR-33a and mir-200c on HFCS-induced fatty liver and vitamin D_3_ supplementation-related liver biochemical and histopathological changes.

## 2. Materials and methods

### 2.1. Characteristics of animals

In this study, 24 male Sprague–Dawley albino rats at 8 weeks, weighing 200–220 g, were used. Animals were fed ad libitum and housed in steel cages having a temperature-controlled environment (22 ± 2 °C) with 12-h light/dark cycles. The sample size was calculated as a minimum of 5 rats per group with Type 1 error α = 0.05 and study power 1-β = %80 using MedCalc® Statistical Software (MedCalc Software Ltd, Ostend, Belgium). All experimental procedures were ethically approved by the University of Health Sciences, Hamidiye Animal Experiments Local Ethics Committee, Turkey. Moreover, National Guide for the Care and Used of Laboratory Animal Experiment Guidelines was followed.

### 2.2. Experimental protocol

#### 2.2.a. Beginning

The study was started when the animals were 8 weeks old (day 0 of the study).

#### 2.2.b. First 8 weeks

Rats were divided into two groups. The first group included 8 rats and was called sham (healthy control) group. This group was given a normal diet. There were 16 rats in the other group and the animals in this group were fed with HFCS.

#### 2.2.c. 8–12 weeks

The first group, in other words the healthy control group, continued to be fed with a normal diet (Group 1). Rats in the experimental fatty liver group were divided into 2 groups. One of them included 8 rats. In this group (Group 2), in addition to the HFCS, rats were given drinking water with an orogastric tube (equal to the amount of vitamin D_3_ replacement with orogastric tube in the next group) weekly for 4 weeks. The last group (Group 3) included 8 rats. They were fed with HFCS, and they were also given 10 μg/kg/week of vitamin D_3_ (Devit, DEVA, Kocaeli, Turkey) by orogastric tube weekly for 4 weeks. In order to induce experimental fatty liver disease model, 35% HFCS was added to drinking water.

At the end of the 12th week, all animals were anesthetized by intraperitoneal (ip) injection of ketamine (40 mg/kg) and xylazine (4 mg/kg) for sacrifice, then intracardiac blood samples were taken, laparotomy was accomplished, and liver tissues were obtained.

### 2.3. Biochemical analysis

The blood samples were obtained after the sacrifice procedure, centrifuged at 4000 rpm for 15 min at room temperature and the serums were separated. All blood samples were stored at −80 °C until the biochemical analysis. Tumor necrosis factor (TNF)-alpha, aspartate aminotransferase (AST), alanine aminotransferase (ALT), and malondialdehyde (MDA) levels were measured with an ELISA device (BioTek Epoch 2 Microplate Spectrophotometer and ELx50 Microplate Strip Washer, Santa Clara, USA) using enzyme-linked immune sorbent measurement (ELISA) kits (Fine Test, Wuhan Fine Biotech Co., Wuhan, Hubei, China). All measurements were made following the instructions included in the study kits.

### 2.4. Histopathological evaluation

#### 2.4.a. Light microscopic study

For histochemical and immunohistochemical studies, rat liver tissue samples were perfused with 200 mL of 4% formaldehyde in 0.1 M phosphate-buffer saline (PBS). After routine procedures, 5-μm-thick liver tissue sections were obtained from formalin-fixed paraffin-embedded tissues with Leica-RM2145 microtome (Nussloch, Germany). All of the stained sections were photographed with Olympus DP72 digital camera mounted on Olympus BX51 microscope (Tokyo, Japan). Slides chosen at random were performed blind microscopic analysis by three histologists at different times/noncontemporary.

#### 2.4.b. Histochemical evaluation

Liver tissue sections were stained with Hematoxylin and Eosin (H&E) (Dako, Glostrup, Denmark) as routine; periodic acid-Schif (PAS) (bio-Optica, Milano, Italy) and Mallory Trichrome (bio-Optica, Milano, Italy) as recommended by the manufacturer’s instructions. Liver damage was examined including the criteria of macrovesicular steatosis, microvesicular steatosis, hepatocyte hypertrophy, and inflammation based on the study by Liang et al. [15]. Briefly macrovesicular steatosis, microvesicular steatosis and hepatocyte hypertrophy criteria were grouped as steatosis meanwhile steatosis and inflammation were evaluated together within NAFLD scoring system for rodents listed in Table 1. One from each block at random, a total of 8 H&E stained sections from each group were selected. Five randomly selected areas were scored as a percentage over 40× (total magnification 400×) magnification. The “percentage of the total area affected” value was obtained by taking the average of these percentage scores recorded at different times by 3 different observers. The severity of adiposity was determined by performing histological steatosis staging (Grade 0-1-2-3) over these percentage values obtained by dividing the whole area (Table 1).

#### 2.4.c. Immunohistochemistry protocol

For immunohistochemistry, sections were incubated with H_2_O_2_ (10%) for 30 min to eliminate endogenous peroxidase activity and blocked with 10% normal goat serum for 1 h at room temperature. Subsequently, sections were incubated in primary antibodies, Ki-67 (Bioss, Inc., Massachusetts, USA, 1/100), iNOS (Santa Cruz Biotechnology, Inc., Texas, USA, 1/100) for 24 h at 4 °C. Antibody detection was performed with the Histostain-Plus Bulk kit (Bioss, Inc., Massachusetts, USA) against rabbit IgG, and 3,3’ diaminobenzidine (DAB) was used to visualize the final product. All sections were washed in PBS and photographed. The slides used in immunohistochemical evaluation were selected at random by three blind observers. Observers examined Ki-67 and iNOS stained slides, eight per both, from each experimental group. One hundred cells were counted at 40× magnification (total magnification 400×). The number of cells that reacted positively was noted, while the latter score was obtained by taking the average. Based on this, immunohistochemical positivity was evaluated as follows: Score 0 (–): 0–5 cells with positive reactions, Score 1 (+): 5–15 cells with positive reactions, Score 2 (++): 15–25 cells with positive reactions, Score 3 (++): 26 and more cells with positive reactions.

### 2.5. RNA extraction from serum samples

The miRNeasy serum/plasma kit (QIAGEN, Hilden, Germany) was used to perform RNA isolation. Two hundred microliters of serum were mixed by vortexing with 1 mL of QIAzol Lysis Reagent. After incubation at room temperature for 5 min, 200 μL of chloroform was added to the homogenized sample, vigorously vortexed, and incubated at room temperature for 3 min. After centrifugation at 12000 × *g* at 4 °C for 15 min, upper aqueous phase were taken to a new collection tube and 100% ethanol was added. Samples were then transferred to RNeasy MinElute spin column. RNA was precipitated with 750 μL of ethanol, triple washed with RPE-buffer, followed by RNA-elution in 20 μL of nuclease-free water, and storage at −80 °C.

### 2.6. Quantitative RT-PCR

Quantitative RT-PCR was performed for the analysis of miRNA expression. cDNA was synthesized using the Taqman miRNA Reverse Transcription Kit (Thermo Fisher, MA, USA). It was then PCR-amplified using Taqman Master Mix (Thermo Fisher, MA, USA). RNU6B was used internally. miRNA levels were quantified by Roche Real-Time 480 PCR (Roche, CA, USA). Relative mRNA expression levels were calculated by the 2 − ΔΔCt method.

### 2.7. Statistical analysis

InStat3 GraphPad Statistics Software (trial version) and IBM SPSS Statistics 18 software were utilized for all tests. Visual (histogram and probability graphs) and analytical methods were used for predicting normal distribution. Normally distributed data in this study were expressed as mean ± SD. Analysis of variance (ANOVA) was used for multiple group comparisons. Those with p-values of <0.05 were considered statistically significant. When a significant difference was noticed between study groups, post hoc tests were performed. qRT-PCR results were analyzed with one way ANOVA test.

## 3. Results

### 3.1. Biochemical results

After one-way ANOVA with Bonferroni post hoc test was performed on biochemical results, statistically significant differences were detected. When the research groups were examined in terms of AST and ALT levels, there were significant differences between the HFCS-induced fatty liver group and vitamin D_3_ supplementation group (p < 0.001). Moreover, there was a significant difference in serum MDA levels between the fatty liver group and vitamin D_3_ group (p < 0.01). On the other hand, there was a statistically significant difference in serum TNF-alpha levels between the experimental NAFLD group and vitamin D_3_ groups (p < 0.01) (Table 2).

### 3.2. Histopathological examination results

**Figure 1:**
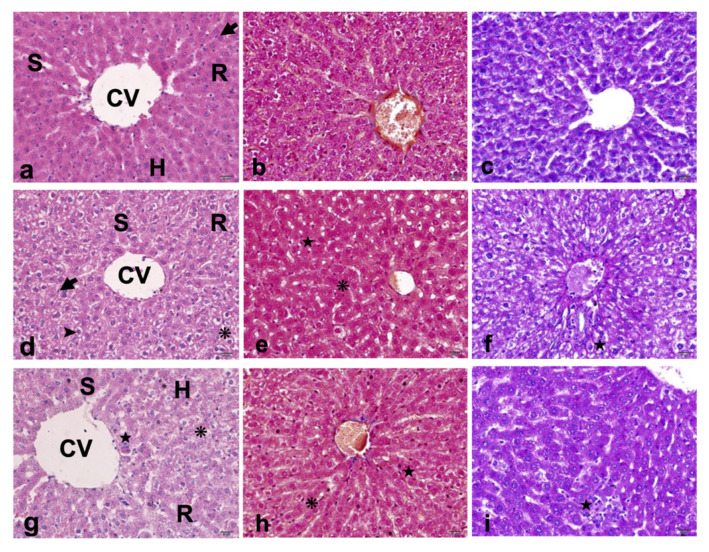
a–c. Normal liver morphology with central vein, well-organized sinusoids and remark cords. Hepatocytes revealed normal cell shape with centrally placed nucleus and eosinophilic cytoplasm. Normal Kupffer cells. No increase in the connective tissue. Homogeneous staining of normal glycogen distribution. d–f. Disorganized liver architecture. Dilated sinusoids, dispersed remark cords and inflammatory cells infiltration were detected. Fatty degenerations as microvesicular steatosis in the hypertrophic hepatocytes with granular cytoplasm and Kupffer cells are indicated. No significant increase in the connective tissue was confirmed. Presence of glycogen deposition was confirmed by strong positive PAS reaction. g–i. Partially protected liver architecture. Reduced microvesicular steatosis, and hepatocellular hypertrophy. Pronounced inflammatory cells infiltration. Mild activation of Kupffer cells. Scale bar = 20 μm, total magnification 400×. *CV: central vein, S: sinusoids, R: remark cords, H: hepatocytes, arrow: Kupffer cells, arrowhead: microvesicular steatosis, asterisk: hepatocellular hypertrophy, star: inflammatory foci*.

In examination of Group 1 slides, stained with H&E, Mallory Trichrome and PAS, normal appearances of the histological structure of liver tissues were observed (Figures 1a–1c). General histological observations consisted of vena centralis located at the center of classical liver lobe, hepatocytes with central-settled nucleus and eosinophilic cytoplasm, neatly lined remark cords around vena centralis and portal areas, and regular sinusoidal structures. Mallory trichrome staining showed no increase in connective tissue. Glucogen distribution among the hepatocytes with PAS staining was normal. No obvious infiltration of inflammatory cells, fibrosis or necrosis was observed, with the exception of minor hepatic steatosis. Liver lobules of experimental fatty liver group were disordered. Dispersed remark cords, sinusoidal dilation, inflammatory cell infiltration, hypertrophic hepatocytes filled with multiple fat droplets with central nuclei (microvesicular hepatic steatosis) and hepatocytes filled with large fat droplet with peripheral nuclei (macrovesicular hepatic steatosis) were detected in liver sections of fatty liver group (Figures 1d–1f). NAFLD scores were significantly higher in Group 2 rats. No significant connective tissue increase or fibrosis was observed with stained Mallory Trichrome sections in Group 1. Hepatocytes of Group 2 rats were stained by PAS, which indicated the presence of glycogen around the central vein and portal area. Liver architecture integrity of the vitamin D_3_ group (Group 3) was partially protected (Figure 1g–1i). A dispersed distribution of hepatocytes with fewer lipid droplets per cell was observed in Group 3. Moreover, microvesicular steatosis and hepatocellular hypertrophy were reduced in Group 3. In addition, NAFLD scores were considerably reduced in Group 3 compared to Group 2. On the other hand, pronounced inflammatory cell infiltration and mild activation of Kupffer cells were observed around hepatocytes and portal area. In the vitamin D_3_ supplementation group, the distribution of PAS stained cells was closer to that of the control (Table 3).

### 3.3. Immunohistochemistry results

**Figure 2:**
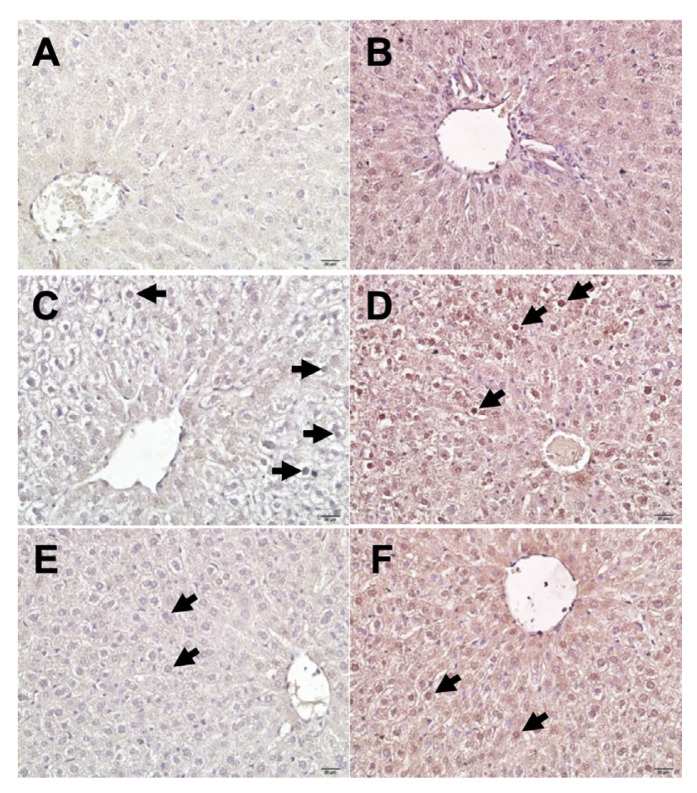
Immunohistochemical stainings of different groups. Ki-67 (left column) and iNOS (right column) immunostained sections of Group 1 to 3, from up to down, respectively. A,B: Barely detectable immunopositivity; C,D: strong positive expression; E,F: relatively weak expression. Black arrow indicates positive expression of immunohistochemical staining. Scale bars = 20 μm, total magnification 400×.

**Figure 3:**
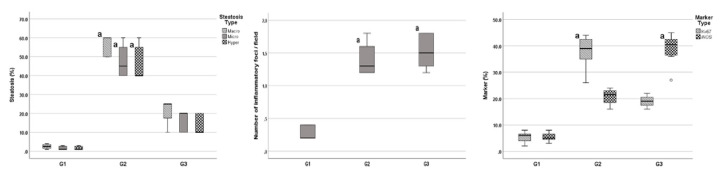
Histopathological results of study groups (macrovesicular steatosis, microvesicular steatosis, hepatocellular hypertrophy, inflammation, and Ki-67 and iNOS, respectively).

In the sham group, liver sections showed negative immunochemical reaction of Ki-67 (Figures 2A and 2B). Liver specimens of Group 2 exhibited strong positive expression of Ki-67 (Figures 2C and 2D). The expression of Ki-67 was considerably reduced in Group 3 compared to Group 2 (Figures 2D and 2E). The expressions of iNOS were hardly detected in the control group. In contrast, it was increased significantly in Group 2 and iNOS immunoreactivity was observed around central vein and in cytoplasm of hepatocytes. We observed that iNOS expression in Group 3 is similar to that in Group 2 (Figure 3) (Table 4).

### 3.4. Quantitative RT-PCR and in silico analyses results

**Figure 4:**
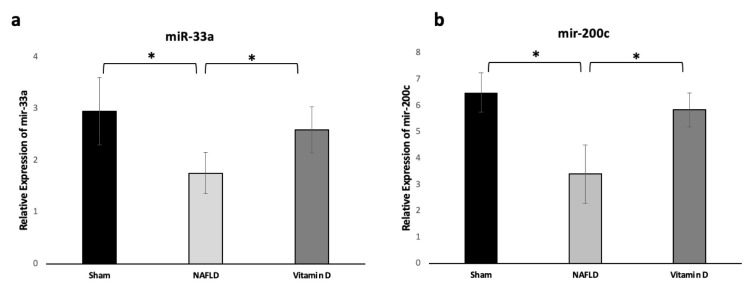
Expression levels of mir-33a (a) and mir-200c (b) among study groups. NAFLD: Nonalcoholic fatty liver disease *p < 0.05.

After the real-time PCR procedure, miR-33a and mir-200c expression levels were found to be significantly decreased in the experimental fatty liver group compared to the control group. Moreover, there was a significant difference in the expression levels of these two miRNAs between the vitamin D_3_ supplementation group and the experimental NAFLD group. Moreover, there was no significant difference between the vitamin D_3_ and sham groups in terms of these two miRNA levels (Figures 4a and 4b).

**Figure 5:**
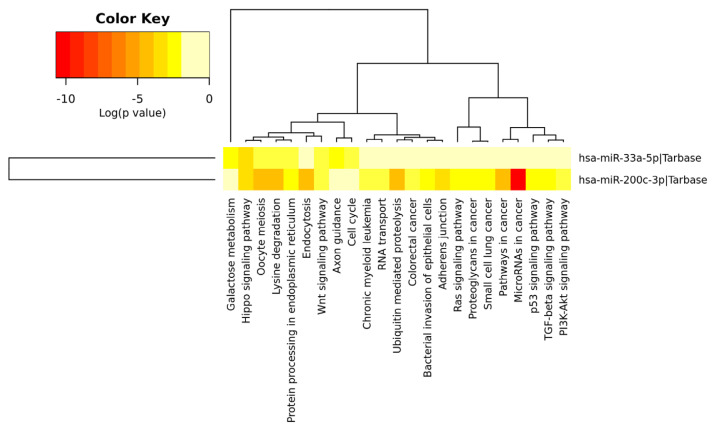
The significant clusters/heat map representation of mir-33a and mir-200c.

As a result of the KEGG pathway analysis, miRNAs (mir-33a, mir-200c) were found to play a common role in the Hippo signaling pathway, lysine degradation, protein processing in endoplasmic reticulum, and Wnt signaling pathway (Table 5) (Figure 5).

## 4. Discussion

Consumption of HFCS products has been strictly integrated to modern western-style diet and excess exposure to HFCS products are one of the prominent factors blamed for body fat accumulation, obesity, and also fatty liver. Clinically acceptable therapeutic agents are being investigated for this health problem [16]. This fact was one of the important trigger points for our current experimental research.

Vitamin D is one of the most investigated topics nowadays, and the number of studies about vitamin D, which is in fact a hormone although historically called a vitamin, will continue increasing. The growing curiosity and evidence about positive effects of vitamin D_3_ to chronic metabolic, cardiovascular, and neoplastic issues have led to widespread utilization of vitamin D_3_ supplementation for the prevention and treatment of numerous disorders [17, 18].

In one huge metaanalysis, it has been reported that higher vitamin D_3_ concentrations were associated with lower likelihood of cardiovascular disease, lipid disorders, glucose metabolism disorders, infectious diseases, multiple sclerosis, mood disorders, and total mortality [19]. Results of our current study suggest that HFCS increases the inflammation and leads to fatty liver in animals and vitamin D_3_ supplementation may have a protective effect against this process. In their study, Roth et al. have demonstrated that vitamin D deficiency, along with a Western-style eating pattern, results in insulin resistance and NAFLD, eventually leading to nonalcoholic steatohepatitis (NASH) (20). However, the authors have provided no data about the effects of vitamin D_3_ supplementation in that study [20].

Vitamin D deficiency is common all over the world and it has been regarded that vitamin D deficiency may be related with NAFLD [21]. Thus, it can be suggested that vitamin D supplementation may prevent or reduce fatty liver development. A few previous studies assessing this assumption have shown both decreased insulin resistance and improved inflammatory markers with vitamin D supplementation [22].

Insulin resistance, diabetes, obesity, and NAFLD are considered chronic inflammatory situations and there are numerous studies demonstrating a correlation between these groups of consequences and TNF-alpha [23, 24]. Moreover, some previous studies have shown that vitamin D supplementation decreases the TNF-alpha expression or at least may induce a clinical improvement in inflammation. For example, Shab-Bidar et al. have found that vitamin D supplementation resulted in a significant improvement in TNF-alpha levels in patients with type 2 diabetes [25]. In contrast, Belenchia et al. have carried out a clinical study on obese adolescents and have reported that vitamin D supplementation resulted in no significant decrease in inflammatory markers despite the decreased insulin resistance [22]. However, in our current study, decreased levels of TNF-alpha in vitamin D_3_ supplementation group support the hypothetical positive role of vitamin D_3_ on hepatic inflammation.

NAFLD is often diagnosed by ultrasonography, which has low sensitivity and specificity. The definitive diagnosis of NAFLD is usually made by liver biopsy, which is an invasive procedure. Due to the limitations of liver biopsy, need for the use of noninvasive markers has emerged in recent years [26]. The presence of miRNAs in body fluids such as serum, plasma, or urine and the fact that they reflect the physiological or pathological conditions of the tissue they belong to provide a great advantage in their use as noninvasive biomarkers [27].

Vitamin D_3_ affects the expression of ZEB1, ZEB2, and SNAI1, which are inducers of epithelial-mesenchymal transition. Relatedly, miR-200b and miR-200c target ZEB1 [28]. Feng et al., in their experimental study in 2014, showed that miR-200c was down-regulated in liver tissues in a NAFLD model created with a high-fat diet [29]. In our current study, unlike Feng et al., we examined the changes in the experimental fatty liver model that we created with a high fructose corn diet. The results of these two studies are similar in terms of showing the relationship between mir-200c and NAFLD. The different and unique aspect of our current study is exhibiting a significant change in circulating mir-200c levels in an experimental fatty liver model. Moreover, vitamin D_3_ supplementation also affected these circulating mir-200c levels [29]. The most important advantage of demonstrating the change in circulating miRNA expression levels in experimental liver studies is not to have to reach the liver tissue in an invasive way.

Erhartova et al. suggested that circulating miR-33a levels may be an indicator of liver steatosis development and inflammation in patients with liver transplantation [30]. In addition, mir-33a plays a critical role in the regulation of fatty acid and cholesterol homeostasis [31]. Moreover, it has been reported that mir-33a is directly related to insulin resistance [32]. For the above reasons, in this current study, we examined the expression level of mir-33a in serum samples in HFCS induced fatty liver model. Finally, we found that circulating mir-33a levels were changed significantly with the development of fatty liver and also with vitamin D_3_ supplementation [30]. Our current results suggest that easily available circulating mir-33a will be used as a biomarker for the diagnosis of fatty liver and also disease course.

As an inevitable finding, in this current study, we showed that liver structure was clearly changed with HFCS. Microvesicular steatosis and sinusoidal dilatation occurred and inflammatory cell infiltration took place. Moreover, it was a very prominent positive finding that vitamin D_3_ supplementation caused less microvesicular adiposity and hepatocellular hypertrophy in hepatocellular zone. Previously, Han et al. created a fatty liver model with a choline-deficient diet and examined the effects on the liver by applying different doses of vitamin D_3_ supplementation [33]. Differently, we showed that fatty liver can easily develop after HFCS application, which is widely consumed in the community. In addition, for the first time in the literature, we regarded that vitamin D_3_-related liver changes are associated with miR-33a and mir-200c, and circulating expression levels of these miRNAs may be diagnostic for fatty liver.

In conclusion, our current fatty liver study demonstrated that vitamin D_3_ supplementation, especially in a proper dose, could attenuate adverse inflammatory, metabolic and hepatic effects of HFCS via miR-33a and mir-200c. Moreover, at a specified dose and at a limited time interval, vitamin D_3_ can diminish HFCS-induced hepatic lipid accumulation, inflammation, and also fatty degeneration. Finally circulating miR-33a and mir-200c may be used as biomarkers for predicting fatty liver and vitamin D_3_ supplementation-related hepatic changes.

## Figures and Tables

**Table 1:** Histological scoring system for NAFLD.

Histological feature	Score 0	Score 1	Score 2	Score 3
Steatosis				
Macrovesicular steatosis	<5%	5%–33%	33%–66%	> 66%
Microvesicular steatosis	<5%	5%–33%	33%–66%	> 66%
Hepatocellular hypertrophy	<5%	5%–33%	33%–66%	> 66%
Inflammation				
Number of inflammatory foci/field	<0.5	0.5–1	1–2	>2

**Table 2:** Biochemical analysis results according to groups.

	Sham	Fatty liver	Vitamin D_3_	p-values
**n**	8	8	8	-
**TNF-** **α** **, pg/mL**	40.1 ± 6.3	53.8 ± 6	44.6 ± 4.9	a0.01
**ALT, IU/L**	50.8 ± 6.4	101.3 ± 11.3	64 ± 9	a0.001
**AST, IU/L**	57 ± 5.5	116 ± 13.4	69.6 ± 8.8	a0.001
**MDA, ng/mL**	26.3 ± 4.8	38.2 ± 6.9	29.6 ± 4.7	a0.01

a One-way ANOVA test with post hoc test (Bonferronni Test). Results are expressed as mean ± standard deviation (SD).

TNF-α: Tumor necrosis factor alpha, ALT: Alanine aminotransferase, AST: Aspartate aminotransferase, MDA: Malondialdehyde.

**Table 3:** Histological scores of rat groups.

Histological feature	Group 1	Group 2	Group 3
Steatosis	Score	Score	Score
• Macrovesicular steatosis	0	1	1
• Microvesicular steatosis	0	2	1
• Hepatocellular hypertrophy	0	2	1
Inflammation	Score	Score	Score
• Number of inflammatory foci/field	0	2	2

**Table 4:** Immunohistochemistry scoring system of iNOS and Ki-67.

Immunohistochemistry scoring system	Group 1	Group 2	Group 3
	Score	Score	Score
• iNOS	0 (−)	2 (++)	2 (++)
• Ki-67	0 (−)	2 (++)	1 (+)

**Table 5:** Overrepresented pathways, which could be affected by miR-33a and miR-200c.

# KEGG pathway	p-value	#miRNAs
Hippo signaling pathway	0.012	2
Lysine degradation	0.004	2
Protein processing in endoplasmic reticulum	0.001	2
Wnt signaling pathway	0.013	2

## Data Availability

The datasets used and/or analyzed during the current study are available from the corresponding author on reasonable request.
